# Cereal biomass-derived xylo- and malto-oligosaccharides as next-generation prebiotics: a comprehensive review of sustainable production and translational benefits for human and animal gut microbiota

**DOI:** 10.3389/fmicb.2026.1803157

**Published:** 2026-03-20

**Authors:** Anel Kostanova, Yuliya Shamsiyeva, Azamat Yermukhanov, Assel Kiribayeva

**Affiliations:** 1 National Center for Biotechnology, Astana, Kazakhstan; 2 Institute of Engineering and Food Technology, S.Seifullin Kazakh Agrotechnical Research University, Astana, Kazakhstan

**Keywords:** cereal biomass, enzymatic hydrolysis, malto-oligosaccharides, prebiotics, xylo-oligosaccharides

## Abstract

Next-generation prebiotics are increasingly recognized for their ability to modulate gut microbiota and promote host health. Xylo-oligosaccharides (XOS) and malto-oligosaccharides (MOS), derived from cereal biomass, are particularly promising due to their structural stability, low effective dosages, and selective stimulation of beneficial gut microorganisms. This review synthesizes current knowledge on XOS and MOS, with emphasis on enzyme-enabled production technologies, microbial fermentation behavior, and health outcomes in humans and animals. Recent advances in tailored enzyme cocktails and sequential bioconversion strategies enable precise control over oligosaccharide yield and degree of polymerization, facilitating targeted production of functionally distinct fractions. Comparative analyses demonstrate that XOS and MOS offer greater microbial selectivity than conventional prebiotics, such as fructo- and galacto-oligosaccharides, and consistently enhance short-chain fatty acid production. These effects contribute to improved metabolic regulation, immune function, and resistance to enteric pathogens. A distinctive contribution of this review is the valorization of Kazakhstan’s underutilized cereal residues, including wheat, rice, barley, and millet straw and bran, as sustainable feedstocks for prebiotic production. Integrating regional biomass with engineered enzyme systems provides a scalable and environmentally sound platform. Furthermore, combined XOS–MOS formulations may exert synergistic microbiota-modulating effects through complementary fermentation dynamics. Collectively, these insights position enzyme-based conversion of cereal biomass as a novel, regionally relevant strategy for advancing next-generation prebiotics.

## Highlights

- Xylo-oligosaccharides (XOS) and malto-oligosaccharides (MOS) derived from cereal biomass are emerging next-generation prebiotics with high physicochemical stability and low effective dosage.
- Enzyme-assisted bioconversion of lignocellulosic and starch-rich residues enables sustainable and scalable production of XOS and MOS.
- Combined XOS–MOS formulations may provide complementary fermentation along the colon and enhance short-chain fatty acid production.

## 1 Introduction

Prebiotics are defined as “a substrate that is selectively utilized by host microorganisms conferring a health benefit” (Gibson et al., 2017). In the past two decades, the prebiotic concept has evolved beyond conventional carbohydrate-based substrates to include next-generation oligosaccharides engineered for enhanced selectivity, increased structural stability, and targeted modulation of the gut microbiota (de Sousa et al., 2025; González-Alonso et al., 2026). Among these, xylo-oligosaccharides (XOS) and malto-oligosaccharides (MOS) have garnered significant scientific and industrial attention.

Compared to traditional prebiotics such as fructo-oligosaccharides (FOS) and galacto-oligosaccharides (GOS), XOS and MOS demonstrate superior physicochemical stability. They are effective at lower dosages and exhibit high resistance to acidic and enzymatic degradation in the upper gastrointestinal tract (Samanta et al., 2015; Palaniappan et al., 2021; Valladares-Diestra et al., 2023). These attributes facilitate their selective fermentation in the colon, thereby enhancing functional efficacy and broadening their application in both human and animal nutrition.

XOS are short-chain oligomers composed of xylose units linked by β-(1 → 4)-glycosidic bonds, typically exhibiting a degree of polymerization (DP) between 2 and 7 (de Gruening Mattos et al., 2024). This molecular structure enables preferential utilization by beneficial intestinal bacteria, such as *Bifidobacterium* and *Lactobacillus* species, resulting in increased short-chain fatty acids (SCFAs) production and improved intestinal homeostasis (Baker et al., 2021; Chen et al., 2021a; Yang et al., 2015). MOS are α-(1 → 4)-linked glucose oligomers, including maltotriose, maltotetraose, and maltopentaose, which also promote bifidogenic activity and stimulate SCFA production, as demonstrated in various *in vitro* and *in vivo* studies (Yin et al., 2019; Jang et al., 2020; Tang et al., 2022). Altogether, these features establish XOS and MOS as promising next-generation prebiotics.

The biological significance of XOS and MOS is increasingly supported by evidence linking their intake to improvements in glucose and lipid metabolism, modulation of inflammatory responses, and enhanced antioxidant defenses. Recent studies also indicate protective effects against obesity-related metabolic disturbances, colorectal dysfunction, and neurological processes associated with the gut-brain axis (Rivière et al., 2016; Markowiak and Śliżewska, 2017; Zmora et al., 2019; Cronin et al., 2021).

In animal nutrition, dietary supplementation with XOS and MOS enhances feed conversion efficiency, growth performance, gut integrity, and resistance to enteric pathogens. These benefits position XOS and MOS as sustainable alternatives to antibiotic growth promoters (Rastall and Gibson, 2015; Chen et al., 2021b; Xiong et al., 2024).

From a production perspective, XOS are predominantly sourced from hemicellulose-rich cereal biomass, including wheat straw, barley straw, rice husks, oat hulls, and cereal bran fractions (OECD-FAO, 2022; Yu et al., 2026). MOS, in turn, are produced from starch-containing cereal substrates, such as wheat bran, rice bran, and other milling by-products that retain residual starch following grain processing (Yang et al., 2026). Utilization of these feedstocks aligns with sustainable bioprocessing, residue valorization, and circular bioeconomy principles.

Kazakhstan holds a particularly advantageous position in this context. As a major cereal-producing country, it generates substantial volumes of agricultural residues each year. A significant portion of this cereal biomass remains underutilized, offering considerable potential for conversion into high-value functional ingredients.

Kazakhstan’s diverse cereal biomass and advancing enzyme biotechnology capabilities provide a competitive advantage for scalable XOS and MOS production. This advantage is particularly notable in comparison to large-scale biomass valorization strategies in the United States, China, and Turkey (Pandey and Sharma, 2024; Ntunka et al., 2025).

Recent progress in enzyme engineering and microbial biocatalysis has expanded the feasibility of large-scale oligosaccharide production. Selective conversion of cereal bran and straw into XOS and MOS can be achieved with tailored xylanolytic and amylolytic enzyme systems. Process efficiency, however, is determined by factors such as enzyme family selection, substrate architecture, degree of polymerization control, and sequential hydrolysis strategies. Regional research increasingly demonstrates the technical potential of these enzymatic platforms, reinforcing Kazakhstan’s capacity to contribute to next-generation prebiotic biomanufacturing (Bouiche et al., 2023).

Despite recent advancements, existing reviews remain fragmented, typically addressing either technological aspects of oligosaccharide production or biological health outcomes, without integrating enzymatic mechanisms, industrial scalability, regional biomass availability, and translational relevance within a unified framework. Therefore, this review provides a comprehensive and integrative assessment of xylo- and malto-oligosaccharides, encompassing raw material selection, production technologies, structural and functional properties, microbiota-mediated mechanisms, and health effects in humans and animals. Particular emphasis is placed on the valorization of cereal biomass and on Kazakhstan’s agro-industrial potential as a sustainable foundation for next-generation prebiotic development.

Despite significant progress in the development of xylo- and malto-oligosaccharides, several critical research gaps continue to limit their translational and industrial implementation. At the production level, standardized enzymatic protocols that generate well-defined, reproducible degree of polymerization (DP) profiles are lacking. This is a major bottleneck, as DP strongly affects microbial selectivity and biological efficacy. This limitation complicates the interpretation and comparison of biological studies. Consequently, well-designed randomized controlled trials in humans evaluating XOS, MOS, and conventional prebiotics under harmonized experimental conditions remain limited (Hermiati et al., 2024).

Furthermore, although both oligosaccharides exhibit individual prebiotic activity, the potential synergistic effects of combined XOS-MOS formulations remain underexplored, particularly regarding microbiota cross-feeding, spatial fermentation dynamics along the colon, and maintenance of microbial diversity. These biological uncertainties are further compounded by the lack of scalable techno-economic assessments. Life-cycle considers that accounts for region-specific cereal biomass supply chains. Together, these gaps highlight the need for an integrated framework that links enzymatic design, structural control, microbiota functionality, and industrial feasibility.

This review integrates enzymatic, mechanistic, and translational dimensions, with particular consideration of regional biomass availability, thereby providing a holistic roadmap for future research.

In comparison to previous reviews that focus on single oligosaccharide classes, the present review integrates microbial amylolytic and xylanolytic strategies for the selective production of both MOS and XOS from structurally distinct cereal by-products (Samanta et al., 2015).

## 2 Production of XOS and MOS from cereal biomass

### 2.1 Raw materials: cereal biomass and industrial by-products

The availability and choice of raw materials are decisive for the efficiency, economic feasibility, and environmental sustainability of xylo-oligosaccharides (XOS) and malto-oligosaccharides (MOS) production. Among renewable resources, cereal biomass represents one of the most abundant and strategically important carbon reservoirs for the development of next-generation prebiotics (Kumar et al., 2024).

Xylo-oligosaccharides are predominantly produced from lignocellulosic substrates rich in hemicellulose, particularly arabinoxylans. Agricultural residues from cereal cultivation, including wheat, barley, oat, rye, and millet straw, as well as rice husks and cereal processing by-products such as bran, are widely recognized as promising feedstocks for XOS synthesis (Amorim et al., 2019a; Dyshlyuk et al., 2024; de Freitas et al., 2019). These materials are inexpensive, renewable, and largely underutilized, especially in grain-producing regions of Central Asia (Askarova et al., 2022).

In contrast, malto-oligosaccharides are obtained from starch-containing cereal resources. Wheat, maize, rice, barley, sorghum, oats, and millet are the main starch-rich substrates used for MOS production (Tetlow and Emes, 2017; Punia, 2020). The efficiency of MOS formation strongly depends on starch composition, including the amylose-to-amylopectin ratio, granule morphology, and gelatinization properties. These factors influence enzymatic accessibility and hydrolysis kinetics (Wang Y. et al., 2022).

At the industrial level, the most economically viable strategies rely on valorizing surplus biomass streams generated by the agro-food sector. These include cereal straw and bran from the processing of wheat, rice, barley, oats, millet, and rye, which provide continuous, regionally concentrated raw material supplies (Skendi et al., 2020). Utilization of such by-products supports circular bioeconomy principles by reducing waste, minimizing environmental impact, and converting low-value residues into functional ingredients with high market potential (Nargotra et al., 2024).

Kazakhstan and the broader Central Asian region possess significant competitive advantages in this context due to their extensive grain production systems. The country annually generates large volumes of cereal-derived residues, notably wheat and barley straw, rice husks, and millet stalks. These residues remain largely underexploited for the manufacture of high-value bioproducts (Food and Agriculture Organization of the United Nations, 2025). These lignocellulosic materials typically contain approximately 25–35% cellulose, 20–30% hemicellulose, and 10–15% lignin. Hemicellulose is particularly rich in xylans, which are the primary precursors for XOS synthesis (Campos Vega et al., 2020; Kumari et al., 2024).

According to national agricultural statistics and international databases, Kazakhstan produces approximately 13–14 million tons of wheat straw, nearly 4 million tons of barley straw, about 1.5 million tons of rice husks, and around 0.8 million tons of millet residues per year (OECD-FAO, 2024; World Bank, 2025). Additional by-products come from oats, rye, triticale, and buckwheat cultivation (Mishra et al., 2024; Toplicean and Datcu, 2024). A substantial proportion of this biomass is currently burned in fields or left to decompose, causing environmental pollution resulting in a significant loss of economic and biochemical potential.

In parallel with lignocellulosic resources, Kazakhstan also generates considerable quantities of starch-rich cereal crops. Wheat dominates national production, while rice, barley, oats, millet, and sorghum provide additional starch-containing feedstocks suitable for MOS synthesis (Food and Agriculture Organization of the United Nations, 2023). The total annual starch potential of cereal biomass exceeds several million tons, providing a robust foundation for large-scale oligosaccharide production.

MOS are produced by enzymatic hydrolysis of gelatinized starch, with product profiles determined by the starch structure and the enzyme specificity. Variations in amylose content, crystalline structure, and granule size significantly affect hydrolysis efficiency and the distribution of malto-oligosaccharides with different degrees of polymerization (Punia, 2020; Xin et al., 2024). Wheat- and rice-derived starch fractions, including those present in bran-rich streams, therefore represent valuable supplementary substrates for MOS generation.

However, several challenges remain, including heterogeneous biomass composition, seasonal variability, high ash and moisture content, and limited infrastructure for centralized collection and preprocessing. Addressing these constraints will require developing regional biomass hubs and integrating oligosaccharide production facilities into existing grain-processing industries.

The availability and composition of cereal-derived biomass resources in Kazakhstan relevant for the production of xylo- and malto-oligosaccharides are summarized in Table 1. The table highlights both lignocellulosic residues rich in hemicellulose, which serve as precursors for XOS, and starch-rich cereal materials suitable for MOS production.

**Table 1:** Major agricultural residues and starch-containing crops in Kazakhstan are relevant to the production of XOS and MOS.

Raw material type	Example crops/residues (Kazakhstan)	Estimated annual availability (million tons)	Main component for prebiotic production	Potential product	References (DOI)
Cereal biomass	Wheat straw, barley straw, rice husks, millet stalks, oat and rye straw, triticale residues, buckwheat straw	Wheat ~13.5; Barley ~4.0; Rice ~1.5; Millet ~0.8	Hemicellulose (xylans, 20–30%)	XOS	Dyshlyuk et al. (2024) Askarova et al. (2022) Nargotra et al. (2024)
Starch-rich cereal resources	Starch-rich cereal crops (wheat, maize, rice, barley, sorghum)	Wheat grain ~14.5; Maize ~6.0; Rice ~1.5; Wheat bran ~2.0–2.5; Other cereal brans (barley, oat) ~ 0.5–1.0	Starch (amylose/amylopectin)	MOS	Food and Agriculture Organization of the United Nations (2023) Xin et al. (2024)

In Kazakhstan, abundant cereal residues, including straw and bran, offer significant potential for the production of XOS and MOS. Understanding their structural and compositional features is essential for efficient enzymatic hydrolysis.

### 2.2 Structural features of cereal bran and straw affecting enzymatic hydrolysis

The efficiency of enzymatic conversion of cereal biomass into MOS and XOS is strongly influenced by the structural organization of cereal bran and straw. Key determinants include the architecture of plant cell walls and the physicochemical interactions between starch and hemicelluloses. The presence of lignin, particularly in straw tissues, and the pronounced structural variability among cereal species also play an important role. Collectively, these factors regulate enzyme accessibility, hydrolysis kinetics, oligosaccharide yield, and the resulting degree of polymerization (DP), ultimately influencing functional prebiotic properties (Wang et al., 2020).

Cereal bran and straw have type II primary cell walls, consisting of cellulose microfibrils embedded in a complex matrix of hemicelluloses, predominantly arabinoxylans, along with β-glucans and phenolic compounds. In cereal bran, the relatively low lignin content and higher tissue porosity generally favor enzymatic hydrolysis. However, extensive substitution of the xylan backbone with arabinose residues and ferulic acid–mediated cross-linking can significantly restrict endo-xylanase activity, limiting XOS release and influencing DP distribution (He et al., 2021).

Interactions between starch and arabinoxylans represent an additional structural constraint in cereal bran. Residual starch granules can form physical associations with hemicellulosic polymers through hydrogen bonding and surface adsorption, reducing enzymatic accessibility of both substrates. These interactions may slow hydrolysis rates and promote the formation of mixed MOS/XOS fractions, complicating precise DP control. Consequently, the sequential or coordinated application of amylolytic and xylanolytic enzyme systems is often required to achieve selective oligosaccharide production (Vangsøe et al., 2021).

In cereal straw, lignin is the main factor conferring recalcitrance to biomass. Lignin forms a dense hydrophobic barrier around polysaccharide fibers and participates in lignin-carbohydrate complexes via covalent linkages to xylans. This architecture severely limits enzyme penetration and may lead to non-productive adsorption of hydrolytic enzymes, thereby reducing catalytic efficiency. As a result, enzymatic production of XOS from straw typically requires prior physicochemical pretreatment to disrupt lignin-polysaccharide associations and improve substrate accessibility (Kim et al., 2025).

Structural variability among cereal species further modulates enzymatic hydrolysis behavior. Wheat and rye are particularly rich in arabinoxylans and are therefore well suited for XOS production. In contrast, oats and barley contain high levels of β-glucans, which affect system viscosity and influence enzyme diffusion. Maize and rice straw exhibit higher lignification degrees, requiring more intensive pretreatment strategies. This interspecies heterogeneity highlights the importance of cereal-specific process design and tailored enzyme systems to optimize MOS and XOS yields and structural profiles.

Overall, a detailed understanding of cereal bran and straw architecture is essential for rational development of bioprocesses aimed at the controlled enzymatic generation of functional oligosaccharides with targeted prebiotic activity.

### 2.3 Chemical structure of xylo-oligosaccharides

Xylo-oligosaccharides (XOS) are short-chain carbohydrates composed of D-xylose residues linked mainly by β-(1 → 4)-glycosidic bonds. They are produced through partial enzymatic or controlled acid hydrolysis of xylan, the main hemicellulosic polysaccharide of cereal cell walls. The degree of polymerization of XOS typically ranges from DP2 to DP6, with xylobiose (DP2) and xylotriose (DP3) being the most abundant and biologically active fractions.

In addition to linear structures, XOS may contain substituent groups such as arabinose, acetyl moieties, or ferulic acid residues, forming substituted arabinoxylo-oligosaccharides. These structural features affect solubility, resistance to gastrointestinal digestion, fermentation kinetics, and microbial selectivity. The β-(1 → 4) linkage configuration provides high stability under acidic gastric conditions while allowing selective utilization by beneficial intestinal bacteria, particularly *Bifidobacterium* species.

Recent studies have shown that the structural characteristics of XOS, including DP, degree of substitution, and molecular conformation, critically determine their prebiotic efficacy and their profiles of short-chain fatty acid production (Chen et al., 2021b; Huang et al., 2022).

**Figure 1:**
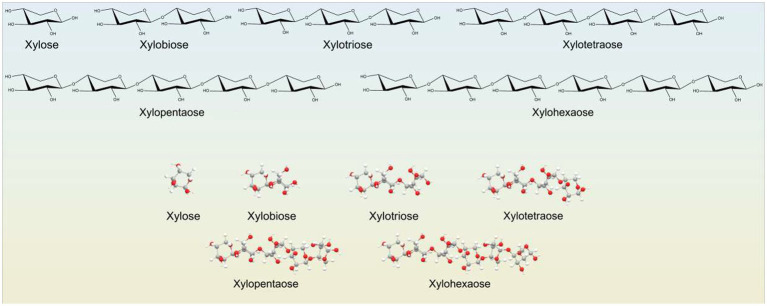
General chemical structure of xylo-oligosaccharides derived from cereal arabinoxylans.

The structural organization of xylo-oligosaccharides, including the β-(1 → 4)-linked xylose backbone and the variations in degree of polymerization, plays a key role in their physicochemical properties and prebiotic functionality. A generalized representation of XOS molecular structures derived from cereal arabinoxylans is shown in Figure 1.

### 2.4 Chemical structure of malto-oligosaccharides

Malto-oligosaccharides (MOS) are linear α-glucans composed of D-glucose units linked by α-(1 → 4)-glycosidic bonds. They are produced by partial enzymatic hydrolysis of gelatinized starch using α-amylase, β-amylase, or related amylolytic enzymes. The degree of polymerization typically ranges from DP2 to DP10, with maltose (DP2) and maltotriose (DP3) being the predominant fractions.

The α-(1 → 4) linkage configuration determines the physicochemical properties of MOS, including high aqueous solubility, rapid enzymatic degradability, and controlled fermentation by gut microbiota. Chain length strongly affects metabolic fate: shorter MOS are fermented rapidly in the proximal colon, whereas longer-chain fractions contribute to sustained fermentation.

MOS have attracted increasing attention as functional carbohydrates and emerging prebiotic candidates. They promote the growth of beneficial microbes and can serve as intermediates in synbiotic formulations. Structural features influencing MOS bioactivity have been comprehensively reviewed in recent molecular-level studies (Bláhová et al., 2023).

**Figure 2:**
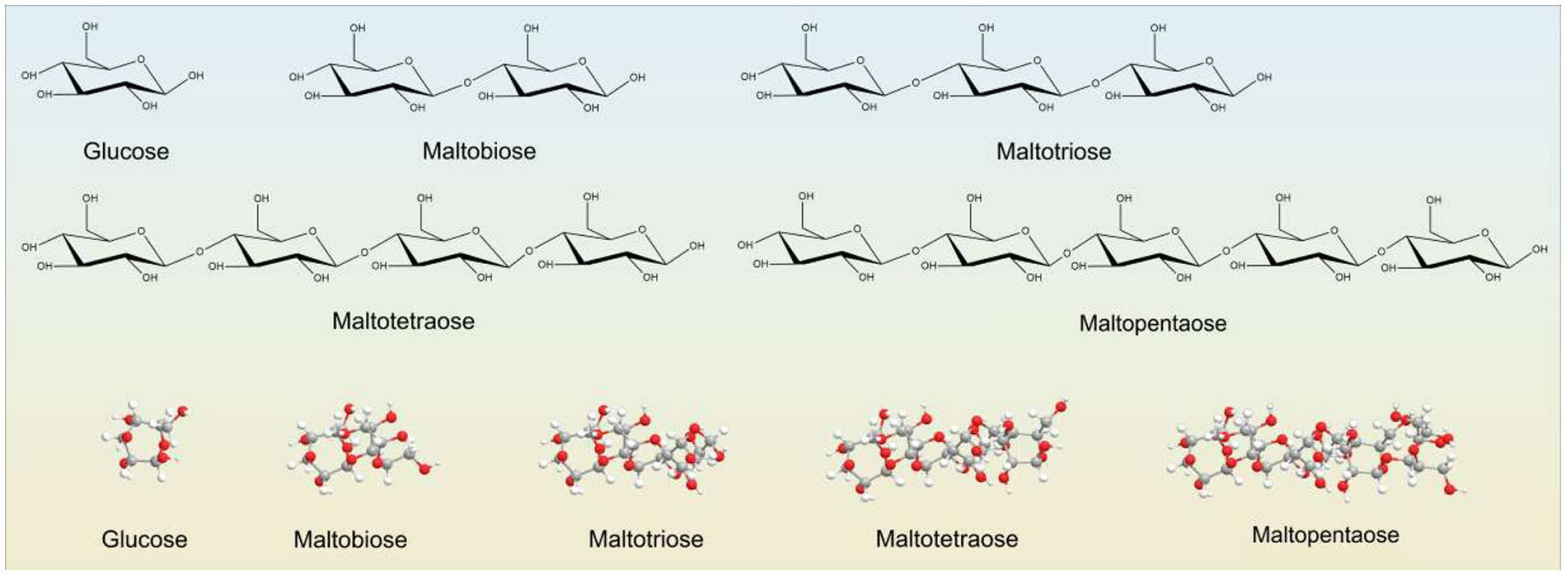
Chemical structure of malto-oligosaccharides (MOS): α-(1 → 4)-linked glucose units with increasing degrees of polymerization (DP).

The molecular structure of malto-oligosaccharides consists of linear α-(1 → 4)-linked glucose units with varying degrees of polymerization, which largely determine their physicochemical properties and fermentability. A general representation of MOS chemical structure is shown in Figure 2.

Overall, the distinct chemical structures of malto- and xylo-oligosaccharides, derived from starch and hemicellulose, respectively, govern their enzymatic accessibility and hydrolysis. These structural differences require tailored pretreatment and enzymatic strategies for cereal bran and straw to enable the controlled production of MOS and XOS with predictable functional properties.

### 2.5 Pretreatment methods

Efficient pretreatment represents a critical prerequisite for the conversion of lignocellulosic and starch-rich cereal substrates into fermentable intermediates suitable for the production of xylo-oligosaccharides (XOS) and malto-oligosaccharides (MOS). The selected pretreatment strategy strongly influences not only oligosaccharide yield but also their structural integrity, degree of polymerization, and functional properties (Huang et al., 2022).

Mechanical processing methods, including milling, grinding, and extrusion, are commonly used as initial pretreatment steps. These approaches increase biomass surface area, reduce particle size, and partially disrupt plant cell wall architecture, thereby improving subsequent enzyme accessibility (Norfarhana et al., 2024). Although mechanical treatments alone are generally insufficient to achieve extensive polysaccharide depolymerization, they are widely used in combination with physicochemical or enzymatic processes to enhance overall hydrolysis efficiency.

In addition, the formation of localized high-temperature zones («hot spots») during microwave treatment facilitates partial deconstruction of crystalline regions within lignocellulosic matrices. This phenomenon significantly enhances enzymatic accessibility and has been shown to increase XOS yield while simultaneously reducing lignin-associated inhibitory compounds. Among emerging technologies, microwave-assisted pretreatment has attracted increasing attention due to its ability to induce rapid volumetric heating and accelerate structural disruption of cereal biomass. Microwave irradiation promotes internal pressure buildup through evaporation of bound water, weakens hydrogen bonding between cellulose and hemicellulose, and reduces polysaccharide crystallinity (Poletto et al., 2020).

As a result, hemicellulosic fractions become more soluble and accessible for downstream enzymatic hydrolysis.

Compared with conventional hydrothermal pretreatment, microwave-assisted systems often require shorter processing times and lower chemical inputs, supporting their potential integration into sustainable biorefinery concepts.

Overall, microwave-assisted pretreatment is a promising approach for improving the efficiency of cereal biomass conversion into functional oligosaccharides. Its effectiveness is particularly pronounced when combined with mild chemical or enzymatic strategies that preserve oligosaccharide structure and biological activity.

Ionic liquids (ILs) are an emerging pretreatment technology capable of efficiently dissolving lignocellulose and starch while preserving fermentable sugars. ILs disrupt hydrogen bonds in cellulose and hemicellulose, improving enzymatic digestibility. However, challenges such as cost, recyclability, and toxicity remain barriers to large-scale application (John and Selvarajan, 2024).

Recent studies have demonstrated the effectiveness of advanced combined pretreatment strategies for enhancing oligosaccharide production from lignocellulosic biomass. In particular, integrated ionic liquid pretreatment with 1-ethyl-3-methylimidazolium acetate, followed by steam explosion, was shown to promote efficient delignification and deacetylation of *Miscanthus* biomass, enabling the selective generation of high-value xylo-oligosaccharides and gluco-oligosaccharides (Bhatia et al., 2021). Such hybrid approaches improve polysaccharide accessibility while limiting excessive carbohydrate degradation, supporting controlled oligosaccharide release.

Acid pretreatment strongly influences xylo-oligosaccharide formation by facilitating partial hydrolysis of hemicellulose, particularly xylan, into oligomeric fractions. The efficiency and selectivity of this process depend on acid type, concentration, reaction temperature, and residence time. Although acid pretreatment offers rapid kinetics and operational simplicity, high acid concentrations often cause over-hydrolysis of xylan into xylose monomers and promote the formation of inhibitory by-products, including furfural and hydroxymethylfurfural (Martins et al., 2023).

To mitigate these limitations, increasing attention has focused on the use of weak or diluted acids (typically <10%), including both organic and inorganic acids. These allow partial depolymerization of hemicellulose while preserving oligosaccharide structures (Hoang et al., 2021). Comparative studies have shown that citric acid outperforms maleic acid in producing XOS from industrial xylan-rich residues, achieving yields of 52.3 and 48.9%, respectively (Madadi et al., 2022).

In addition to Brønsted acids, Lewis acid catalysts have emerged as promising alternatives for hemicellulose depolymerization. These include metal chlorides such as potassium chloride, zinc chloride, ferric chloride, copper (II) chloride, as well as solid acid catalysts. Lewis acids exhibit high catalytic activity, relatively low cost, and reduced toxicity compared with strong mineral acids, making them attractive for environmentally compatible oligosaccharide production (Zhang et al., 2022).

Overall, rational selection and optimization of acidic and hybrid pretreatment strategies are essential for achieving high XOS yields while minimizing inhibitor formation. These approaches provide valuable tools for tailoring oligosaccharide structure and enhancing the sustainability of cereal biomass valorization.

Alkaline pretreatment is regarded as one of the most effective approaches for preparing lignocellulosic biomass for downstream enzymatic conversion. Alkaline reagents disrupt hydrogen bonds and ester linkages between hemicellulose, cellulose, and lignin, promoting hemicellulose solubilization and enhancing substrate accessibility for subsequent hydrolysis (Santibáñez et al., 2021).

A wide range of alkaline agents has been applied for this purpose, including sodium hydroxide (NaOH), ammonia (NH₃), potassium hydroxide (KOH), calcium hydroxide (Ca(OH)₂), and sodium carbonate (Na₂CO₃). These reagents differ in delignification efficiency, carbohydrate preservation, and environmental footprint. Ammonia-based pretreatment, in particular, shows strong selectivity for lignin removal while minimizing polysaccharide degradation.

Several studies have reported successful XOS production from rice straw and rice husk following ammonia pretreatment and enzymatic hydrolysis, confirming their suitability for hemicellulose valorization (Khat-udomkiri et al., 2018). Optimized alkaline pretreatment conditions have also been described for soft wheat bran. Treatment at 15% solid loading using 0.08 mol·L^−1^ NaOH at 150°C for 20 min resulted in a total sugar concentration of 101.29 g·L^−1^ after enzymatic hydrolysis (Zhao et al., 2020).

In practical biorefinery systems, integrating multiple pretreatment strategies has been shown to enhance process efficiency further while reducing chemical consumption and environmental impact. For example, combinations of microwave irradiation with enzymatic hydrolysis or mechanical disruption followed by ionic liquid treatment have been reported to increase carbohydrate accessibility and sugar yields (Bhatia et al., 2021).

Moreover, autoclave-assisted steam treatment under acidic conditions has been shown to effectively release fermentable sugars, including total sugars, xylose, and glucose, from liquid hydrolysates while simultaneously modifying the residual solid fraction to enhance its enzymatic digestibility (Debiagi et al., 2020).

Overall, alkaline and hybrid pretreatment strategies represent powerful tools for improving lignocellulosic deconstruction and tailoring oligosaccharide production. Their rational integration within bioprocessing schemes enables higher XOS yields, improved sugar recovery, and increased flexibility for the valorization of cereal-derived biomass.

The development of scalable, eco-friendly, and cost-effective pretreatment methods is therefore essential for industrial-scale production of XOS and MOS.

**Figure 3:**
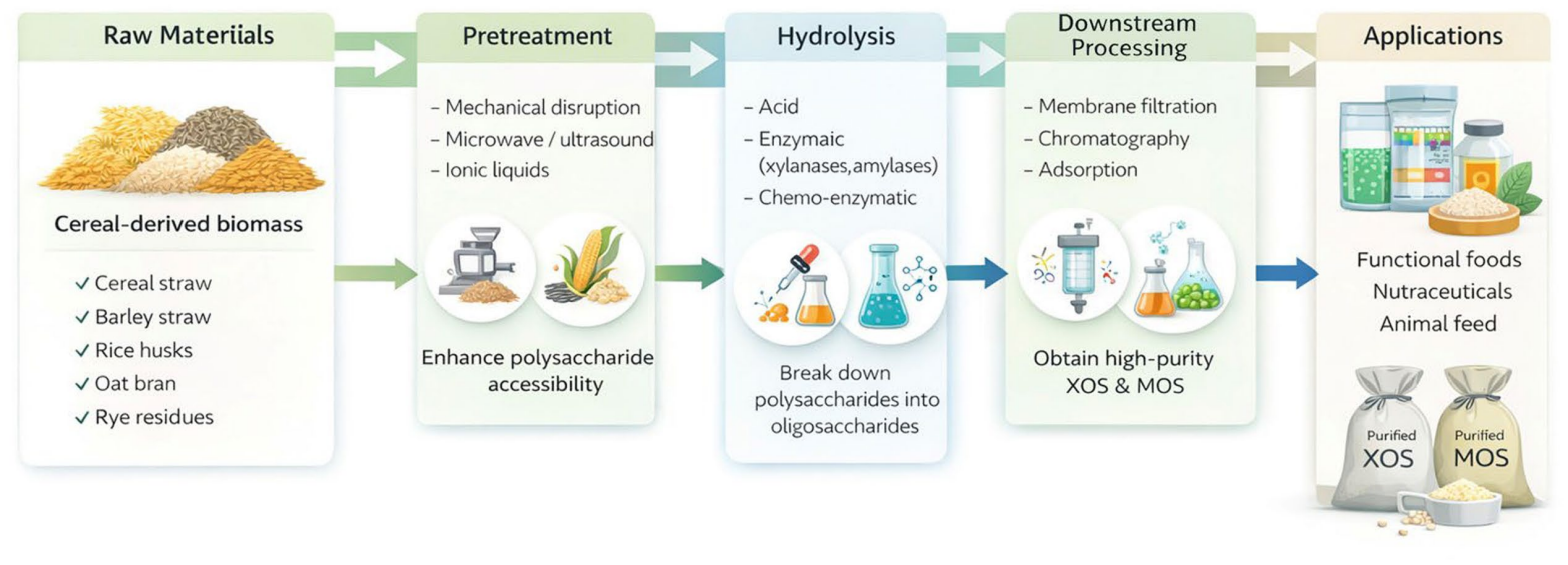
Pretreatment strategies for cereal biomass valorization.

A more detailed overview of the production process is provided in Figure 3, showing specific pretreatment methods (mechanical, microwave, ultrasonication, ionic liquids), hydrolysis approaches (acid, enzymatic, chemo-enzymatic), and downstream purification technologies.

Cereal by-products are subjected to mechanical size reduction and mild alkaline pretreatment to improve polysaccharide accessibility. Following starch gelatinization, amylolytic hydrolysis produces malto-oligosaccharides (MOS, DP 2–6). After solid–liquid separation, the hemicellulose-enriched residue undergoes xylanase-based hydrolysis, yielding xylo-oligosaccharides (XOS, DP 2–6, including arabinoxylo-oligosaccharides). This sequential strategy enables the selective recovery of both oligosaccharide fractions from cereal biomass.

### 2.6 Hydrolysis methods

Following pretreatment, the hydrolysis step converts lignocellulose and starch into oligosaccharides, thereby defining the yields and functional profile of XOS and MOS. Several hydrolysis strategies have been established, ranging from classical chemical methods to advanced enzymatic and hybrid approaches (Saini et al., 2022).

Acid hydrolysis has historically been used for oligosaccharide production, where dilute or concentrated mineral acids (e.g., H₂SO₄, HCl) cleave glycosidic bonds in hemicellulose and starch (Chen et al., 2024). Despite its effectiveness, this approach suffers from limited selectivity and poor control over the degree of polymerization, often leading to excessive depolymerization into monosaccharides. While effective, acid hydrolysis is often accompanied by undesirable by-products (furfural, hydroxymethylfurfural) that can inhibit microbial fermentation and reduce product safety (Li et al., 2013). High acid consumption, equipment corrosion, and wastewater generation further constrain its industrial and sustainable application.

Alternative chemical and physicochemical methods, including hydrothermal, alkaline, and hybrid pretreatment-hydrolysis strategies, have been explored to mitigate these drawbacks. However, these approaches still face challenges in achieving precise structural control over oligosaccharide profiles. Consequently, enzymatic hydrolysis has emerged as the preferred strategy for XOS and MOS production, offering higher selectivity, milder processing conditions, and improved control over DP and functional properties.

#### 2.6.1 Enzymatic hydrolysis of lignocellulosic biomass (cereal straw)

Enzymatic hydrolysis is one of the most selective and environmentally friendly approaches for converting lignocellulosic biomass derived from cereal straw into functional xylo-oligosaccharides (XOS). In contrast to acid or thermochemical treatments, enzymatic processes operate under mild conditions (pH 4.0–7.0; 30–60°C) and do not generate toxic degradation by-products such as furfural or hydroxymethylfurfural, which can negatively affect downstream processing and biological activity.

The enzymatic depolymerization of xylan, the major hemicellulosic component of cereal straw, is primarily mediated by the synergistic action of endo-1,4-β-xylanases (EC 3.2.1.8) and β-xylosidases (EC 3.2.1.37). Endo-1,4-β-xylanases cleave internal β-(1 → 4)-glycosidic linkages within the xylan backbone, leading to the formation of short-chain xylo-oligosaccharides with varying degrees of polymerization (DP), including xylobiose (DP2), xylotriose (DP3), and higher oligomers (DP4–DP6), which exhibit pronounced prebiotic activity (Ali et al., 2025).

Xylanases are widely produced by diverse microorganisms, including bacteria such as *Bacillus* spp. and *Streptomyces* spp., as well as filamentous fungi such as *Trichoderma* spp. and *Aspergillus* spp. These enzymes differ substantially in catalytic efficiency, substrate specificity, and tolerance to process conditions, strongly influencing XOS yield and DP distribution. Reported optimal operating conditions for endo-xylanases typically fall within pH 4.0–6.5 and 40–80°C, depending on enzyme origin and structural family.

The physicochemical properties of xylanases also vary considerably. Molecular weights of these enzymes commonly range from approximately 8.5 to 85 kDa, while isoelectric points (pI) span from 4.0 to 10.3, reflecting substantial structural diversity among enzyme families (Choudhury et al., 2025). These variations influence enzyme–substrate interactions, adsorption on lignocellulosic surfaces, and overall hydrolysis performance.

Importantly, enzymatic hydrolysis enables controlled production of XOS with defined DP profiles, which is critical for tailoring prebiotic functionality. Short-chain XOS (DP2–DP3) are preferentially utilized by *Bifidobacterium* and *Lactobacillus*, whereas longer oligomers contribute to prolonged fermentation and increased short-chain fatty acid (SCFA) production in the distal colon. Therefore, the rational selection of enzyme systems and reaction conditions is essential to optimize both the yield and the functional quality of XOS derived from cereal straw. A broad diversity of microbial xylanolytic systems has been reported for the selective production of xylo-oligosaccharides from lignocellulosic substrates. These enzymes differ substantially in glycoside hydrolase (GH) family, microbial origin, substrate specificity, optimal operating conditions, and resulting degree of polymerization of XOS. Representative examples of microbial xylanases used for XOS production are summarized in Table 2.

**Table 2:** Microbial xylanolytic systems and XOS production.

Enzyme (GH family)	Microbial source	Substrate	Optimal working (hydrolysis) conditions	Main products XOS DP	References (DOI)
Endo-β-1,4-xylanase	*Aureobasidium pullulans* var. *melanigenum* CCT 1261	Xylan	50°C, pH 4.5	DP2, DP3,	Corrêa Junior et al. (2022)
Endo-β-1,4-xylanase (GH 11)	*Aspergillus niger* BCC14405	Beechwood xylan	45° C, pH 6.0,	DP2, DP3, DP6	Aiewviriyasakul et al. (2021)
Xylanase	*Acinetobacter pittii* MASK 25	Rice straw Corn cob	40°C, pH 5.0	DP5, DP6	Purohit et al. (2017)
Xylanase (GH10)	*Aspergillus niger* JL15	Beechwood xylan	40°C, pH 5.0	DP2-DP6	Zhang et al. (2024)
Endo-1,4-β-xylanases (GH10)	*Cohnella* sp. strain AR92	Xylan	49.9 to 50.4°C, pH optima from 6.01 to 6.31	DP2, DP3	Hero et al. (2021)
Xylanase XynB	*Penicillium janthinellum* XAF01	Corncob bran	50°C, pH 4.3	DP2, DP3	Zhou et al. (2021)
Xylanase	*Bacillus altitudinis* XYL17	Beechwood xylan Corncob xylan	50–55°C, pH 6.5	DP2, DP3	Phukon et al. (2025)
Xylanase	*Thermomyces dupontii* J22	Corn cobs	60°C, pH 7.0	DP2, DP3	Qiu et al. (2025)
Xylanase	*Trichoderma reesei*	Xylan	42.5°C, pH 6.0	DP5	Amorim et al. (2019b)
Endo-xylanase (GH 11)	*Trichoderma* sp. strain TP3-36	Beechwood xylan	55°C, pH 5.0	DP2, DP3	Fu et al. (2019)
Xylanases	*Aspergillus flavus* KUB2	Xylan	55°C, pH 5.0	DP2-DP5	Supmeeprom et al. (2024)

#### 2.6.2 Enzymatic hydrolysis of starch (MOS)

For MOS, α-amylases catalyze the hydrolysis of α-(1 → 4)-glycosidic bonds, yielding maltotriose, maltotetraose, and higher malto-oligosaccharides (Shinde and Vamkudoth, 2022). Recent enzyme engineering strategies, including thermostable variants and immobilized enzyme systems, have further improved malto-oligosaccharide-forming amylases.

Malto-oligosaccharide-forming amylases (MFAses) constitute a specialized group of glycoside hydrolases, predominantly from the GH13 family. These enzymes catalyze the hydrolysis of α-(1 → 4)-glycosidic bonds in starch and maltodextrins, resulting in the preferential formation of malto-oligosaccharides (MOS). Unlike conventional α-amylases, which primarily generate glucose and maltose, MFAses exhibit pronounced product specificity toward specific oligosaccharide fractions.

This enzyme group includes maltotriose-forming amylase (EC 3.2.1.116), maltotetraose-forming amylase (EC 3.2.1.60), maltopentaose-forming amylase (EC 3.2.1.-), maltohexaose-forming amylase (EC 3.2.1.98), and several related enzymes with similar product selectivity. Collectively, MFAses can produce MOS with degrees of polymerization typically ranging from DP2 to DP10, depending on enzyme origin, substrate structure, and reaction conditions.

The catalytic behavior of MFAses is highly diverse. It may involve endo-type cleavage, exo-type hydrolysis, or so-called multiple-attack mechanisms that combine features of both endo- and exo-action modes. This mechanistic flexibility enables efficient depolymerization of starch granules and maltodextrins, but it also complicates precise control of the final MOS profile. As a result, the distribution of MOS fractions is strongly influenced by enzyme structure, substrate accessibility, and hydrolysis kinetics (Jaafar et al., 2021).

MFAses exhibit activity toward a wide range of starch-based substrates, including gelatinized starch, liquefied starch, and maltodextrins, resulting in the formation of heterogeneous MOS mixtures. Therefore, optimization strategies often rely on careful selection of enzyme type, reaction temperature, pH, and substrate concentration to obtain targeted DP distributions suitable for prebiotic applications.

A broad diversity of bacteria and filamentous fungi from different ecological niches has been identified as natural producers of MFAses. These microorganisms exhibit substantial variability in substrate specificity, catalytic efficiency, thermostability, and product spectrum, providing valuable opportunities for enzyme screening, protein engineering, and tailored MOS production for functional food and feed applications. A wide diversity of microbial amylolytic enzymes has been reported for the production of malto-oligosaccharides with defined degrees of polymerization. These enzymes differ markedly in microbial origin, substrate specificity, optimal reaction conditions, and product profiles, collectively determining the efficiency and selectivity of MOS synthesis. Representative examples of malto-oligosaccharide-forming amylases reported between 2018 and 2025 are summarized in Table 3.

**Table 3:** Microbial amylolytic enzymes for MOS production.

Enzyme (GH family)	Microbial source	Substrate	Optimal working (hydrolysis) conditions	Main products MOS DP	References (DOI)
α-amylase AmyEs (GH13)	*Enhygromyxa salina*	Corn bran	–	DP6	Zhong et al. (2025)
Maltooligosaccharide-forming amylase BkAmy (GH13)	*Bacillus koreensis* HL12	Cassava starch	40°C, pH 7.0	DP2–DP4	Lekakarn et al. (2022)
α-amylase PfAmy and TeAmy	*Pyrococcus. furiosus, Thermococcus eurythermalis*	Starch	90°C for TeAmy, 100°C for PfAmy, pH 5.5	DP2–DP6	Liao et al. (2023)
Amylosucrase CC-ASase + α-amylase PM-Amy	*Cellulomonas carboniz* (CC-ASase) *Pseudomonas mendocina* (PM-Amy)	Sucrose	Sequential enzymatic strategy: CC-ASase (2.5 unit/mL) at 40°C for 4 h, PM-Amy (1.25 unit/mL) at 55°C for 4 h	DP2–DP6	Zhu et al. (2018)
Starch-degrading enzyme	*Priestia koreensis* HL12	Cassava starch, rice starch	65°C, pH 6.0	DP2, DP3, DP5	Lekakarn et al. (2025)
Maltooligosaccharide-forming amylase BstMFAse	*Bacillus stearothermophilus* STB04	Corn starch	60°C, pH 5.5	DP5, DP6	Xie et al. (2019)
Malto-oligosaccharide-forming Amylase AmyCf	*Cystobacter* sp. strain CF23	Starch	pH 7.0 at 60°C for gelatinized starch or 50°C for raw starch	DP2–DP4	Wang et al. (2023)
Amylase AmyAc	*Archangium* sp. strain AC19	Starch	50°C, pH 7.0	DP2, DP3	Fan et al. (2021)
α-amylase AmyM	*Corallococcus* sp. strain EGB	Starch	50°C, pH 7.0	DP6	Li et al. (2015)

The rational selection of enzymatic strategies is a critical determinant of the efficiency of converting cereal biomass into malto- and xylo-oligosaccharides (MOS and XOS), influencing product yield, degree of polymerization (DP) distribution, and functional properties. Contemporary biocatalytic processes employ both sequential and co-treatment approaches, frequently integrated with physicochemical pretreatment and multienzyme systems to enhance substrate accessibility and hydrolysis selectivity.

Simultaneous enzymatic treatment of cereal-derived polysaccharides is based on the synergistic action of multiple enzymes operating concurrently within a single reaction system. This approach reduces processing time and enhances oligosaccharide yields by coordinating the depolymerization of structurally interconnected polymers.

#### 2.6.3 Sequential and integrated enzymatic strategies

For xylan depolymerization, endo-β-1,4-xylanases are commonly combined with accessory enzymes, including α-L-arabinofuranosidases, acetylxylan esterases, and feruloyl esterases. These auxiliary enzymes remove side-chain substitutions and ester-linked phenolic crosslinks, thereby increasing the accessibility of the xylan backbone and promoting the controlled release of xylo-oligosaccharides with defined DP profiles (McCleary et al., 2015; Alokika, 2019; de Mello Capetti et al., 2023).

In parallel, starch hydrolysis is mediated by coordinated endo- and exo-acting amylolytic enzymes, including malto-oligosaccharide-forming amylases (MFAses), which preferentially generate MOS fractions rather than complete saccharification to glucose. Such co-treatment systems enable partial and simultaneous conversion of starch and hemicellulose components within cereal bran, supporting integrated MOS–XOS production platforms.

Sequential enzymatic processing is a highly effective approach for selectively generating prebiotic oligosaccharides from cereal bran and straw. This strategy is based on the stepwise hydrolysis of structurally distinct polysaccharide fractions within lignocellulosic biomass, allowing enhanced control over product composition and minimizing undesired monosaccharide formation.

In the first stage, starch-rich fractions are selectively hydrolyzed following starch gelatinization using amylolytic enzymes, including MFAses, to produce malto-oligosaccharides with targeted DP distributions. Subsequently, the solid residue, enriched in hemicellulosic components, is subjected to xylanase-mediated hydrolysis, leading to the formation of xylo-oligosaccharides (Morais et al., 2023; Leaerts et al., 2024).

**Figure 4:**
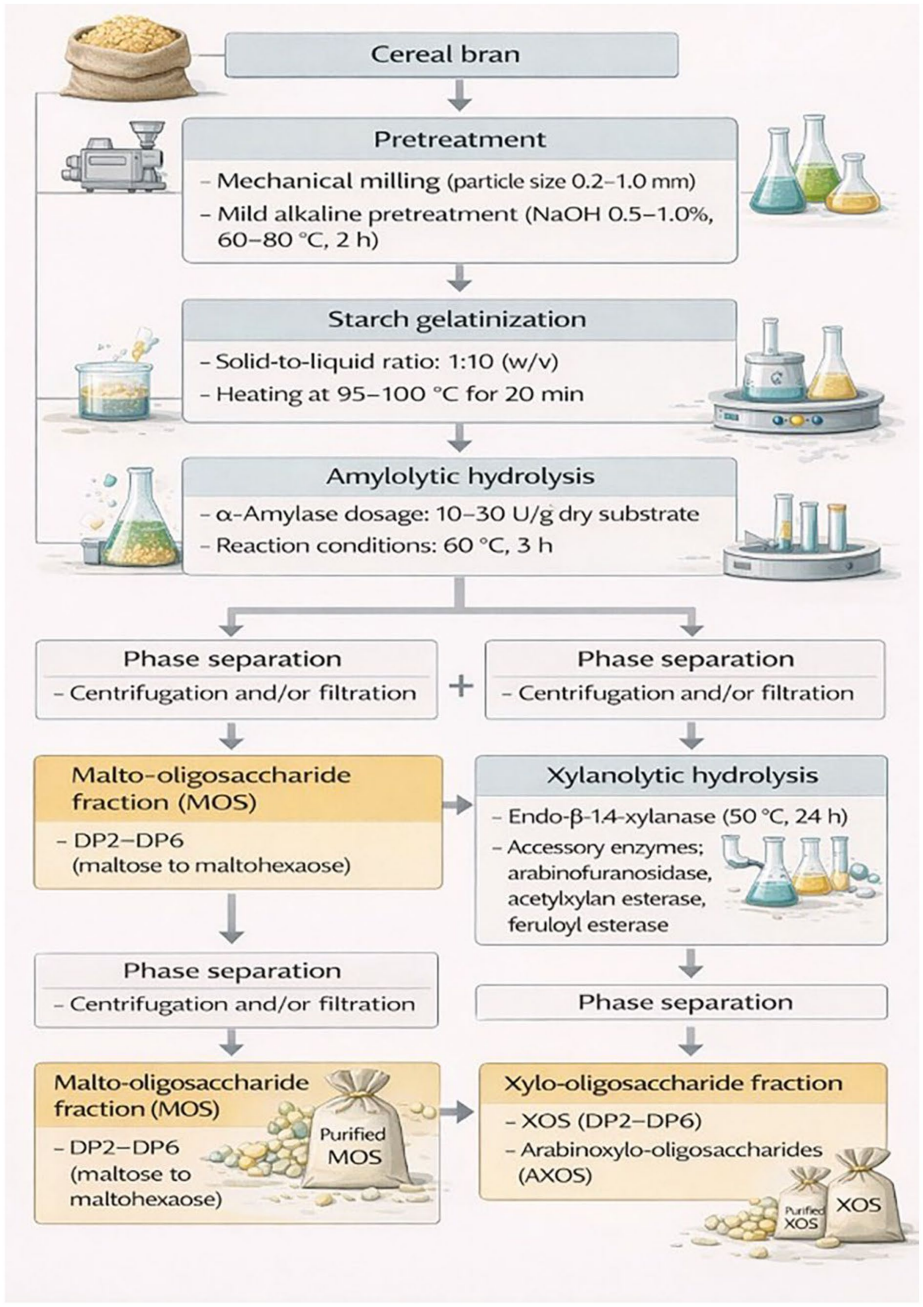
Sequential enzymatic conversion of cereal bran into maltooligosaccharides (MOS) and xylooligosaccharides (XOS).

This sequential enzymatic approach offers several advantages, including improved substrate selectivity, reduced competitive enzyme inhibition, minimized sugar degradation, and enhanced overall valorization of cereal by-products. As a result, it provides a rational framework for integrated bioconversion of cereal bran and straw into high-value prebiotic ingredients. The integrated sequential process involving amylolytic and xylanolytic hydrolysis steps is schematically illustrated in Figure 4.

Combined chemo-enzymatic approaches integrate mild chemical pretreatments (e.g., dilute acid, steam explosion) with enzymatic hydrolysis to balance yield and product purity (Hassan et al., 2025). This hybrid strategy reduces enzyme consumption and minimizes inhibitor formation, while ensuring high-quality oligosaccharides.

Collectively, hydrolysis methods determine the molecular weight distribution, degree of polymerization, and bioactivity of XOS and MOS. Advances in enzyme discovery and biocatalysis are likely to replace traditional acid hydrolysis with cleaner, more sustainable processes in industrial settings (Chakraborty and Bhowal, 2025).

As hydrolysis strategies ultimately define the composition and quality of XOS and MOS mixtures, efficient downstream processing and purification are essential for separating target oligosaccharides, removing residual inhibitors, and meeting product specifications suitable for food and feed applications.

### 2.7 Downstream processing and purification

After hydrolysis, the separation and purification of XOS and MOS are critical to achieving products with consistent quality, safety, and functionality in the products. Crude hydrolysates usually contain monosaccharides, degraded polysaccharides, fermentation inhibitors (furfural, phenolics), and salts that must be removed before food or feed applications (de Paula et al., 2025).

Membrane filtration (ultrafiltration, nanofiltration) is widely used to separate oligosaccharides from monosaccharides and other impurities, offering high selectivity, energy efficiency, and scalability (Thiele et al., 2025).

Membrane separation processes are based on the principles of ultrafiltration (UF) and nanofiltration (NF). In these systems, carbohydrates with a higher degree of polymerization are retained in the retentate, whereas monosaccharides and low-molecular-weight impurities pass through the membrane into the permeate. Membrane filtration is characterized by technological simplicity, high scalability, and relatively low energy consumption, making it a highly attractive approach for industrial oligosaccharide purification.

An additional advantage of membrane-based technologies is the ability to immobilize biocatalysts directly on the membrane surface or within the membrane matrix. This configuration enables the development of integrated membrane bioreactor systems that allow continuous synthesis of malto-oligosaccharides (MOS) while simultaneously separating products. Such integration of enzymatic catalysis and mass transfer enhances substrate conversion efficiency, improves overall process performance, increases enzyme stability, and permits repeated enzyme reuse. These features are particularly important for intensifying large-scale oligosaccharide production processes, including MOS (Bláhová et al., 2023).

For the purification of xylo-oligosaccharides (XOS), various process schemes combining ultrafiltration and nanofiltration have been developed, making membrane technologies particularly effective for this class of prebiotics. Ultrafiltration is typically employed to separate XOS from high-molecular-weight components, such as residual polymers and enzymes, while maintaining low energy requirements and strong scalability. The combined use of UF and NF significantly enhances process selectivity and product purity (Singh et al., 2019).

Nanofiltration membranes enable efficient concentration of XOS and effective removal of monosaccharides and other low-molecular-weight compounds (Córdova et al., 2019).

In several studies, a two-stage UF strategy was applied to purify XOS obtained via enzymatic hydrolysis of previously isolated xylan, resulting in effective elimination of sugar monomers and the production of highly purified XOS syrups with degrees of polymerization ranging from DP 2 to DP 5 (Kumar and Satyanarayana, 2015).

In other studies, an ultrafiltration membrane with a molecular weight cut-off (MWCO) of 10 kDa removed high-molecular-weight impurities, including proteins and non-hydrolyzed polysaccharides. This process resulted in a significant enrichment of the XOS fraction (Kammakakam and Lai, 2023).

The permeate obtained after 10 kDa ultrafiltration was subsequently processed using a 1 kDa nanofiltration membrane, which allowed complete removal of xylose. As a result, the final hydrolysates contained XOS with degrees of polymerization between DP 2 and DP 5 and exhibited a purity of approximately 78% (Li et al., 2021).

Differences in separation efficiency across reported studies can be attributed to variations in operational parameters, including cross-flow velocity, temperature, transmembrane pressure, and the pore-size distribution of the selected membranes. Overall, membrane-based purification represents a robust, scalable, and industrially relevant strategy for producing high-purity MOS and XOS suitable for applications in functional foods, nutraceuticals, and animal nutrition.

Chromatographic methods, particularly activated charcoal column chromatography and ion-exchange chromatography, provide high purity but are often cost-intensive and limited to laboratory-scale applications (Kruschitz and Nidetzky, 2020).

Among chromatographic techniques, gel permeation chromatography (GPC), also known as gel filtration chromatography (GFC), has gained widespread recognition as an effective tool for the purification and fractionation of oligosaccharides based on molecular weight. This method has been extensively applied for the isolation of high-purity xylo-oligosaccharides (XOS) and enriched hydrolysates, enabling the separation of fractions with distinct degrees of polymerization (DP) and the removal of low-molecular-weight impurities, such as monosaccharides, organic acids, and degradation products (Lian et al., 2020).

Gel permeation chromatography has also been employed for the fractionation of malto-oligosaccharide (MOS) mixtures. In these applications, polyacrylamide-based gels and hydrophilic vinyl polymer gels have been predominantly used. However, previous studies have demonstrated that such chromatographic systems exhibit limited resolution and low loading capacity, which restricts their applicability for large-scale purification. In particular, the isolation of individual MOS fractions with DP ≥ 6–7 at a preparative scale remains challenging due to insufficient separation efficiency and high operational costs (Bláhová et al., 2023).

In addition to chromatographic approaches, adsorption and precipitation-based techniques have been explored as alternative or complementary strategies for purifying XOS and MOS. These methods rely on the selective interaction between oligosaccharides or accompanying impurities and the surface of solid adsorbents, followed by controlled desorption or elution of target fractions. Various adsorbent materials have been investigated, including titanium dioxide, activated carbon, diatomite, aluminum oxide, bentonite, porous synthetic polymers, silica gels, and hydroxide-based sorbents (Santibáñez et al., 2021).

Among these materials, activated carbon remains one of the most extensively studied and widely applied adsorbents for the purification of both XOS and MOS. It is particularly effective for the removal of colored compounds, phenolic impurities, residual lignin fragments, and monosaccharides from hydrolysates obtained after enzymatic processing of lignocellulosic or starch-rich substrates. Moreover, adsorption processes using activated carbon can contribute to partial fractionation of oligosaccharides based on their degree of polymerization.

The application of 10% (w/v) activated carbon, combined with elution with a 5% ethanol solution, enabled effective purification of XOS, yielding a product purity of approximately 48% while preserving oligosaccharide fractions with DP values ranging from 1 to 9 (Kruschitz and Nidetzky, 2020). Similar adsorption-based strategies have also been reported for MOS purification, particularly for removing monosaccharides, low-molecular-weight sugars, and colored by-products generated during enzymatic starch hydrolysis (Bláhová et al., 2023).

Overall, while chromatographic techniques offer high resolution and structural selectivity, their limited scalability and high operational costs limit their industrial implementation. In contrast, adsorption-based methods offer greater economic feasibility and process simplicity, especially when integrated with membrane technologies in multi-step downstream processing schemes for large-scale XOS and MOS production.

Integrated biorefinery approaches now combine membrane separation with enzymatic treatments and green solvents, reducing process costs and environmental impact while ensuring food-grade quality (Wienberg et al., 2022).

The choice of purification method ultimately depends on the intended application: high-purity XOS/MOS are required for nutraceuticals and pharmaceuticals, while moderate purity is acceptable for animal feed.

Different hydrolysis strategies have been developed to produce XOS and MOS from lignocellulosic and starch-based feedstocks. Each method has distinct advantages and limitations in terms of yield, selectivity, cost, and sustainability. A comparative overview of these production approaches is provided in Table 4.

**Table 4:** Comparison of major production strategies for xylo-oligosaccharides (XOS) and malto-oligosaccharides (MOS).

Method	Substrate	Enzymes/catalyst	yield and efficiency	Advantages	Limitations	References (DOI)
Acid hydrolysis	XOS: lignocellulosic biomass; MOS: starch	Dilute H₂SO₄, HCl	Moderate yield, DP not well controlled	Simple, scalable, low enzyme cost	Harsh conditions, by-product formation (furfural, HMF), low selectivity	Martins et al. (2023)
Enzymatic hydrolysis	XOS: xylan-rich hemicellulose; MOS: starch	Xylanases, β-xylosidases, α-amylases	High yield, controlled DP	High selectivity, mild conditions, preserves functionality	High enzyme cost, enzyme inhibition possible	Marim and Gabardo (2021)
Combined chemo-enzymatic	Same as above	Mild acid + enzymes	High yield and selectivity	Balances cost and efficiency	More complex optimization needed	Grootaert et al. (2007)
Green pretreatments (microwave, ultrasonication, ionic liquids)	Agricultural residues, starch crops	Physical + enzymatic hydrolysis	Improved accessibility, higher hydrolysis rate	Eco-friendly, reduces chemical use, enhances enzyme action	Scale-up challenges, higher initial costs	Zhai et al. (2024)
Integrated biorefinery	Mixed agricultural residues	Enzyme cocktails, membrane purification	High purity and scalability	Zero-waste approach, aligns with circular economy	Requires advanced infrastructure	Silva et al. (2022)

Table 4 compares the main approaches to producing XOS and MOS, illustrating that enzymatic hydrolysis remains the most efficient and selective, while emerging eco-friendly pretreatments and integrated biorefinery concepts hold promise for scalable, sustainable applications.

Beyond production strategies, a comparative evaluation of XOS and MOS against other commercially relevant oligosaccharides is essential to contextualize their prebiotic performance and application potential.

### 2.8 Comparison with other oligosaccharides (FOS, GOS, AXOS, IMO)

While XOS and MOS are emerging prebiotics, their functional properties should be evaluated against those of other well-established oligosaccharides.

- Fructo-oligosaccharides (FOS) and galacto-oligosaccharides (GOS) are among the most widely studied and commercialized prebiotics. They are recognized by regulatory authorities and supported by extensive evidence for their bifidogenic effects and long-term safety. However, they exhibit broad fermentability, being utilized by a wide range of gut microbes rather than selectively stimulating key beneficial taxa, and typically require higher doses (2.5–10 g/day) to elicit significant effects, whereas XOS and MOS can achieve comparable or stronger prebiotic effects at lower intake levels (1–2 g/day) (de Sousa et al., 2025; Yang et al., 2015; Tang et al., 2022). Recent studies further indicate that specific oligosaccharide types, including XOS, may confer more selective stimulation of *Bifidobacterium* and *Lactobacillus* spp. relative to FOS/GOS (Nogueira-Prieto et al., 2025).
- Arabinoxylan-oligosaccharides (AXOS), derived from cereal hemicellulose, share structural similarities with XOS and have been shown to exhibit bifidogenic activity, increasing the abundance of *Bifidobacterium* in the human gut (Feng et al., 2025). However, comparative studies suggest that XOS may exhibit a slightly greater selective stimulation of *Bifidobacterium* spp., suggesting subtle differences in microbial specificity between these oligosaccharides. This nuanced perspective enables a more accurate assessment of prebiotic potential, while recognizing that both AXOS and XOS contribute to gut health (González-Alonso et al., 2026).
- Isomalto-oligosaccharides (IMO) are produced from starch and exhibit moderate prebiotic effects but often lack the stability and tolerance profiles of MOS (Zhai et al., 2024). IMO, preparations tend to be partially digestible and partially absorbed in the upper gastrointestinal tract, thereby reducing the amount reaching the colon compared with more resistant MOS fractions (Hu et al., 2020).

Comparative analyses consistently indicate that XOS and MOS not only achieve stronger and more targeted bifidogenic effects at lower dosages but also exhibit superior physicochemical stability and gastrointestinal resilience. At the same time, their production from abundant agro-industrial residues enhances sustainability and supports circular bioeconomy principles (Valladares-Diestra et al., 2023).

## 3 Structure–function relationships of XOS and MOS

### 3.1 Molecular determinants of functionality

The biological activity of prebiotics is intrinsically linked to their molecular structure. For xylo- and malto-oligosaccharides, parameters such as degree of polymerization, glycosidic linkage type, and substitution patterns critically determine physicochemical behavior, microbial accessibility, fermentation kinetics, and downstream host responses. This section, therefore, integrates structural determinants with functional and biological outcomes, highlighting the structure–function relationships that underpin the prebiotic efficacy of XOS and MOS.

These structural parameters do not merely define chemical identity but also critically shape solubility, stability, fermentability, and microbial selectivity, thereby governing downstream functional and biological effects. In particular, variations in chain length, branching patterns, and glycosidic bonds influence the resistance of XOS and MOS to acidic and enzymatic degradation in the upper gastrointestinal tract, the rate and site of fermentation in the colon, and the profile of short-chain fatty acids produced.

Collectively, these molecular features define not only the biological performance of XOS and MOS but also provide the foundation for their physicochemical characteristics, which are essential for technological applications and effective delivery to the gut microbiota.

### 3.2 Physicochemical properties

Building on their molecular architecture, the technological applicability and prebiotic performance of XOS and MOS are further governed by their physicochemical properties, including solubility, thermal and acid stability, viscosity, and sweetness profile. XOS and MOS are highly water-soluble and exhibit thermal/acid stability compatible with food processing conditions such as pasteurization, baking, or acidic beverages (Silva et al., 2022).

Compared to FOS and GOS, they impart lower sweetness. They are generally better tolerated at lower effective doses (often 1–2 g/day for XOS in adults), reducing risks of bloating or flatulence at application-relevant levels (Niu et al., 2024). In animal studies, XOS and MOS are typically supplemented at 0.1–2 g/kg body weight per day, depending on species and study design, for durations ranging from 2 to 8 weeks to evaluate physiological, microbial, and metabolic outcomes (Xiong et al., 2024; Youssef et al., 2024). *In vitro* fermentation experiments commonly use concentrations of 0.5–2% (w/v) in growth media, with incubation periods of 12–48 h, to assess microbial utilization and SCFA production (Palaniappan et al., 2021; Cheon et al., 2023). Their available energy is lower than that of digestible sugars because a substantial fraction escapes small-intestinal digestion and is fermented in the colon; thus, they can help modulate the calorie density of formulated foods (Gidley, 2024). In complex matrices (dairy, baked goods), XOS and MOS can also serve as bulking agents and cryo/thermo-protectants for probiotics, thereby improving viability during storage and gastric transit (Wang and Zhong, 2024).

Together, these physicochemical properties interact with molecular determinants to govern how XOS and MOS reach the colon, become accessible to gut microorganisms, and exert selective prebiotic effects. By linking structural features to physicochemical behavior, this integrated perspective underscores how XOS and MOS combine stability, functional performance, and targeted modulation of microbiota, setting them apart from other oligosaccharides in both biological and technological contexts.

### 3.3 Interaction with gut microbiota

Xylo-oligosaccharides (XOS) and malto-oligosaccharides (MOS) selectively modulate gut microbiota composition, although their ecological footprints differ due to substrate specificity and fermentation kinetics. XOS preferentially stimulate bifidobacterial taxa equipped with xylanolytic systems, including *Bifidobacterium adolescentis* and *B. longum* subsp. *longum*, while also being partially depolymerized by *Bacteroides* species via polysaccharide utilization loci. This primary degradation facilitates cross-feeding interactions that support secondary fermenters such as *Eubacterium rectale* and *Faecalibacterium prausnitzii*, enhancing butyrate production (de Gruening Mattos et al., 2024; Rivière et al., 2016; Palaniappan et al., 2021).

In contrast, MOS are efficiently utilized by maltodextrin-adapted bifidobacteria, such as *Bifidobacterium breve*, as well as by lactobacilli, resulting in increased production of acetate, propionate, and butyrate, alongside suppression of opportunistic pathobionts such as Enterobacteriaceae *in vitro* and *ex vivo* fermentation models (Jang et al., 2020; Markowiak and Śliżewska, 2017). Collectively, the fermentation of XOS and MOS lowers luminal pH and promotes the generation of short-chain fatty acids (SCFAs), which act as key metabolic intermediates linking microbial activity to host physiological responses, including modulation of gut barrier integrity and immune function (Cheon et al., 2023; Lee et al., 2023).

Importantly, the ecological outcomes of XOS and MOS fermentation extend beyond taxonomic selectivity and are strongly influenced by structure-dependent fermentation kinetics, which govern both the rate of substrate utilization and the intestinal region in which fermentation predominates. Degree of polymerization (DP), glycosidic linkage type, and substitution patterns collectively determine microbial accessibility, cross-feeding efficiency, and the temporal dynamics of short-chain fatty acid (SCFA) production. Consequently, the interaction of XOS and MOS with the gut microbiota reflects a tightly coupled structure–function relationship that links molecular features to spatial and functional outcomes along the colon.

### 3.4 Functional effects by degree of polymerization

The degree of polymerization critically influences fermentation kinetics and the spatial utilization of XOS and MOS along the gastrointestinal tract. Short-chain fractions (DP2-DP4), particularly XOS with DP2-DP3, are rapidly fermented in the proximal colon, leading to early SCFA release, a decline in luminal pH, and pronounced bifidogenic effects (Ali et al., 2025; Saini et al., 2022; Chen et al., 2021a). Longer-chain XOS and MOS fractions are metabolized more slowly and may persist in distal colonic regions, thereby extending fermentation activity and improving spatial SCFA coverage along the colonic axis (Louis and Flint, 2017; Tang et al., 2022). Structural substitution patterns, such as arabinose side chains or acetylation, further modulate depolymerization rates, extending fermentation windows and promoting cross-feeding interactions that enhance butyrate production (Palaniappan et al., 2021; Louis and Flint, 2017). Substituted XOS fractions limit immediate enzymatic attack, promoting stepwise depolymerization and prolonging fermentation windows. Consequently, low-DP and minimally substituted fractions act as rapid substrates for proximal microbiota, whereas higher-DP and heavily substituted fractions continue to feed distal microbial populations, ensuring sustained SCFA release. This staggered utilization enhances cross-feeding between primary degraders and secondary butyrate producers, fine-tuning the timing, spatial distribution, and composition of SCFA profiles along the colon.

Empirical studies confirm that unsubstituted and arabinose-substituted XOS (AXOS) are generally fermented more rapidly than acetylated or glucuronic-acid-substituted XOS, reflecting structural constraints on enzymatic access and microbial utilization. These differences underpin clear structure–activity relationships that determine both the temporal and spatial SCFA profiles and selectively stimulate bifidobacterial and butyrogenic taxa (Nogueira-Prieto et al., 2025; Wu et al., 2020).

Collectively, these findings define a robust structure–activity framework, in which DP and substitution patterns dictate microbial accessibility, fermentation velocity, spatial persistence along the colon, and temporal SCFA release.

### 3.5 Metabolic effects

The metabolic consequences of XOS and MOS consumption arise as downstream effects of microbial fermentation and associated SCFA signaling pathways. SCFAs modulate glucometabolic control by activating enteroendocrine GLP-1 and PYY secretion, enhancing hepatic lipid oxidation, and regulating adipose tissue lipolysis and thermogenesis (Samanta et al., 2015; Chen et al., 2021b; Cheon et al., 2023; Lee et al., 2023). Preclinical studies consistently demonstrate improved insulin sensitivity, reduced hepatic steatosis, and attenuation of adiposity following dietary supplementation with XOS or MOS, often achieving effects comparable to or superior to those of FOS at lower inclusion levels (Palaniappan et al., 2021; Yang et al., 2015).

In human intervention trials, XOS supplementation has been associated with reductions in total and LDL cholesterol and improvements in glycemic markers in both healthy individuals and metabolically at-risk populations (Feng et al., 2022; Thursby and Juge, 2017). These outcomes align with shifts in microbial fermentation profiles that favor propionate- and butyrate-associated metabolic regulation. Inter-individual variability highlights the influence of baseline microbiota composition and dietary context, underscoring the relevance of precision nutrition approaches. Collectively, structure-dependent fermentation dynamics translate into systemic metabolic effects extending beyond the gut lumen.

### 3.6 Immunomodulatory and anti-inflammatory effects

Beyond metabolic regulation, fermentation-derived SCFAs act as key modulators of mucosal and systemic immune responses. XOS and MOS supplementation have been shown to attenuate epithelial NF-κB activation, promote regulatory T cell differentiation, and enhance barrier integrity through mechanisms that include butyrate-mediated histone deacetylase inhibition and GPR43 signaling (Hansen et al., 2019; Hodgkinson et al., 2023; Pérez-Reytor et al., 2021). These processes result in increased IL-10 production and reinforcement of tight junction proteins, including occludin and claudins.

In models of enteric infection and respiratory viral challenge, dietary fiber-driven SCFA production improves mucosal immunity, enhances neutrophil function, and limits excessive inflammatory responses (Kamikado et al., 2020; Trompette et al., 2018). In livestock systems, supplementation with XOS and MOS reduces intestinal loads of *Clostridium perfringens* and *Escherichia coli*, improves villus-to-crypt ratios, and maintains performance outcomes while reducing reliance on subtherapeutic antibiotics (Chen et al., 2021a; Xiong et al., 2024). Together, these immunomodulatory effects support the role of XOS and MOS as functional alternatives aligned with antimicrobial stewardship strategies.

### 3.7 Antioxidant and anticancer potential

While direct antioxidant activity has been reported for specific XOS and MOS preparations, the predominant anticancer effects appear to be mediated indirectly through microbial fermentation products. In particular, butyrate functions as a class I/IIa histone deacetylase inhibitor, promoting apoptosis, cell-cycle arrest, and differentiation in colorectal cancer cells, while simultaneously supporting epithelial DNA repair mechanisms (Davie, 2003; Donohoe et al., 2012; Carretta et al., 2021). Long-term prebiotic supplementation in rodent models of colorectal carcinogenesis consistently reduces tumor burden and dysplasia, with increased butyrate availability and secondary bile acid modulation implicated as central mechanisms (Caruso et al., 2020).

Although clinical outcome data remain limited, the convergence of mechanistic insights and preclinical evidence supports a plausible oncopreventive role for XOS and MOS. These findings provide a rationale for future targeted human intervention studies to define dose–response relationships and population-specific benefits (O’Keefe, 2016).

### 3.8 Gut-brain axis and neuroprotective effects

SCFAs cross the blood–brain barrier and modulate microglial maturation, neuroinflammation, and neurotransmitter pathways (tryptophan/serotonin, GABA), providing plausible mechanisms by which XOS and MOS may influence cognition and stress responses. In murine studies, prebiotic supplementation improved learning and memory and reduced anxiety-like behaviors, with associated shifts in hippocampal BDNF expression and microglial priming that were linked to SCFA signaling. Early human data suggest potential mood and cognitive benefits from targeted prebiotics; however, well-powered randomized controlled trials are required to establish efficacy, optimal dosing, and population-specific responses (Erny et al., 2015; Dalile et al., 2019; Burokas et al., 2017).

To integrate the structural and functional differences discussed in Sections 3.1–3.4, Table 5 presents a comparative overview of XOS, MOS, and established prebiotics. This summary underscores the distinctive properties of XOS and MOS as next-generation prebiotics, combining structural stability, low effective dosage, and microbiota-driven metabolic effects.

**Table 5:** Comparative structural and functional features of XOS, MOS, FOS, and GOS.

Prebiotic	Typical DP	Structural linkages	Main raw material sources	Stability (heat/acid)	Selective microbial utilization	Main metabolic outcomes	Evidence type	Human dose/duration	References (DOI)
XOS	2–7	β-(1 → 4)-linked xylose	Lignocellulosic residues (wheat straw, corn cobs, rice husks, sugarcane bagasse)	High thermal and acid stability	*Bifidobacterium adolescentis*, *B. longum*, *Lactobacillus* spp.; cross-feeding to butyrate producers	↑ acetate, propionate, butyrate; ↓ pathogenic *Enterobacteriaceae*	Human RCTs, animal and *in vitro* studies	1–4 g/day; 2–8 weeks	Niu et al. (2024) Feng et al. (2022)
MOS	3–7	α-(1 → 4)-linked glucose	Starch-rich crops (maize, potato, rice, wheat)	High solubility; stable under food-processing conditions	*Bifidobacterium breve*, *Lactobacillus* spp.; limited utilization by *Bacteroides*	↑ SCFA production, ↑ microbial diversity; ↓ pathogens	Predominantly *in vitro* and animal studies; limited human evidence	Human data limited	Bláhová et al. (2023) Shinde and Vamkudoth, 2022
FOS	2–10	β-(2 → 1)-fructose	Chicory root, Jerusalem artichoke, enzymatic sucrose conversion	Moderate; partial hydrolysis under acidic conditions	*Bifidobacterium* spp., *Lactobacillus* spp.	↑ acetate, lactate; moderate SCFA production	Multiple human RCTs	5–10 g/day; 2–12 weeks	Tang et al. (2022) Ambrogi et al. (2023)
GOS	2–8	β-(1 → 4)/(1 → 6)-galactose	Lactose transgalactosylation	High stability in milk and infant formulas	*Bifidobacterium infantis*, *B. breve*, *Lactobacillus* spp.	↑ bifidobacteria in infants; improved stool consistency	Strong human evidence	2–8 g/day; 2–16 weeks	Kadim et al. (2025)

Table 5 highlights the structural, metabolic, and clinical characteristics of XOS and MOS relative to well-studied counterparts such as FOS and GOS, including degree of polymerization (DP), linkage types, sources, microbial selectivity, and levels of evidence from *in vitro*, animal, and human studies. Reported human intervention studies vary substantially in supplementation doses (typically 1–4 g/day for XOS) and intervention durations (2–8 weeks), which likely contributes to variability in observed metabolic and microbiota-related outcomes (Feng et al., 2022; Thursby and Juge, 2017). Notably, XOS exhibit superior stability under gastrointestinal conditions and promotes cross-feeding to butyrate-producing bacteria, whereas MOS demonstrate high solubility and strong bifidogenic activity. In contrast, FOS and GOS often require higher dosages to achieve similar metabolic effects.

**Figure 5:**
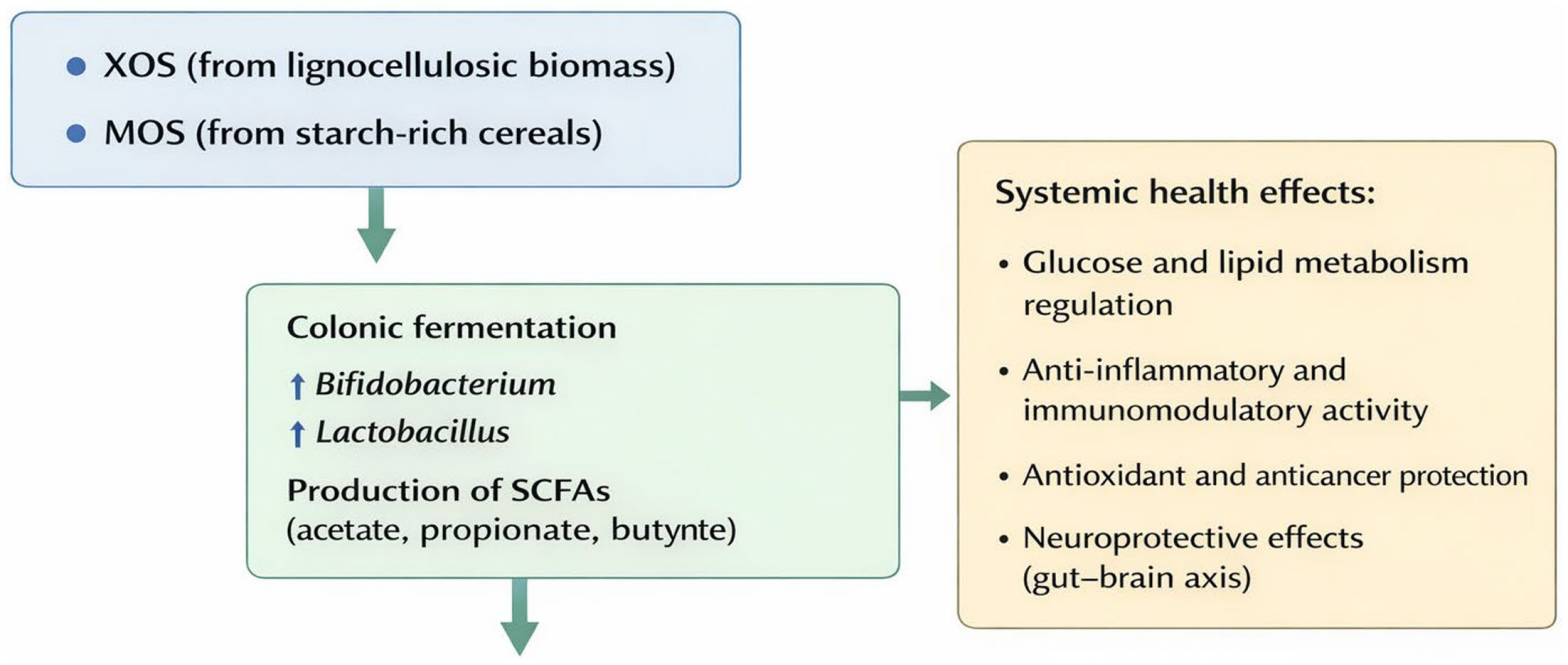
Role of XOS and MOS in gut microbiota modulation and systemic effects.

Collectively, XOS and MOS selectively stimulate beneficial gut microorganisms such as *Bifidobacterium* and *Lactobacillus*, enhancing SCFA production (Figure 5). These metabolites support gut barrier integrity, reduce intestinal inflammation, and mediate systemic effects on glucose and lipid metabolism, immune modulation, and neuroprotection via the gut–brain axis. In summary, these pathways illustrate the multifaceted and complementary roles of XOS and MOS in maintaining host health across metabolic, immune, and neurological domains (de Sousa et al., 2025; González-Alonso et al., 2026).

## 4 Applications in animal nutrition

The use of xylo-oligosaccharides (XOS) and malto-oligosaccharides (MOS) in animal nutrition has expanded rapidly, primarily due to global restrictions on antibiotic growth promoters (AGPs). Instead of directly targeting pathogens, these oligosaccharides enhance host performance by improving intestinal resilience, feed efficiency, and immune competence through microbiota-driven mechanisms described in Section 3 (Chen et al., 2021b; Rastall and Gibson, 2015). This section examines practical outcomes observed in various livestock systems, emphasizing species-specific effects and functional benefits.

### 4.1 Poultry

In poultry production, gut health is a primary determinant of feed conversion efficiency and disease resistance. Dietary supplementation with XOS has been reported to improve intestinal morphology, including increased villus height and villus-to-crypt ratio, thereby enhancing nutrient absorption and growth performance. Consistent increases in beneficial microbial populations are accompanied by reductions in enteric pathogens such as *Clostridium perfringens* and *Salmonella* (Xiong et al., 2024; Wang Q. et al., 2022).

MOS supplementation provides complementary benefits by reducing pathogen adhesion to intestinal epithelial surfaces via mannose-specific lectin interactions (Xin et al., 2024). Across multiple studies, inclusion levels of 0.1–0.2% XOS or MOS have been associated with improved body weight gain, feed conversion ratio, reduced mortality, and enhanced carcass quality, supporting their use as functional feed additives in antibiotic-free poultry systems (Chen et al., 2021a, 2021b).

### 4.2 Swine

In swine production, post-weaning diarrhea and growth suppression remain major challenges. Supplementation with XOS has been reported to improve intestinal environmental stability, increase the availability of fermentative end products, and support beneficial microbial colonization, thereby improving nutrient digestibility and reducing weaning stress (Chen et al., 2021a). MOS supplementation further enhances systemic and mucosal immune responses, lowering the incidence of enteric infections and supporting growth performance (Sun et al., 2025).

Importantly, XOS and MOS supplementation has been shown to improve average daily gain while reducing reliance on zinc oxide and other environmentally unsustainable feed additives, aligning nutritional strategies with evolving regulatory and sustainability demands (Sun et al., 2025).

### 4.3 Ruminants

In ruminant systems, XOS supplementation selectively stimulates fibrolytic microbial populations in the rumen, thereby improving fiber degradation efficiency and volatile fatty acid production. These effects translate into increased milk yield and improved feed utilization in dairy cattle (Li et al., 2026). MOS supplementation has been associated with reduced somatic cell counts, enhanced immune resilience, and improved udder health.

Some studies further report reductions in methane emissions associated with favorable microbial shifts, positioning XOS and MOS as promising nutritional tools aligned with climate-mitigation objectives in ruminant agriculture (Ike et al., 2024).

### 4.4 Aquaculture

In aquaculture, prebiotic supplementation is increasingly used to enhance disease resistance and feed efficiency. XOS supplementation improves gut microbial diversity, enhances nutrient utilization, and increases resistance to bacterial pathogens, including *Aeromonas hydrophila* and *Vibrio* spp. (Swanson et al., 2020). MOS, widely incorporated into shrimp and tilapia diets, enhances innate immune parameters including lysozyme activity, phenoloxidase levels, and phagocytic capacity, resulting in improved survival under pathogen challenge.

Combined XOS-MOS supplementation has demonstrated synergistic effects on growth performance, immune modulation, and survival rates across multiple aquaculture species (Van Doan et al., 2020).

### 4.5 Comparison with antibiotics

Global restrictions on AGPs have created demand for safe, sustainable alternatives. XOS and MOS present advantages over conventional feed additives such as acidifiers, probiotics alone, or high doses of zinc oxide. Unlike antibiotics, these oligosaccharides do not contribute to antimicrobial resistance. Instead, they exert long-term benefits by improving intestinal resilience, nutrient utilization, and overall host performance (Rahman et al., 2022). Their dual role in both microbiota modulation and immune enhancement positions them as next-generation feed additives in modern animal production. Several studies have demonstrated the beneficial impacts of XOS and MOS supplementation across multiple livestock species, including poultry, swine, ruminants, and aquaculture. These outcomes span from improved gut microbiota balance and immune modulation to enhanced growth performance and feed efficiency. Representative findings are summarized in Table 6.

**Table 6:** Performance of XOS/MOS in livestock nutrition.

Animal species	Supplement (dose)	Main outcomes	References (DOI)
Broiler chickens	XOS 0.5–1.0% in diet	↑ Bifidobacterium and Lactobacillus counts, ↓ pathogenic bacteria, ↑ feed conversion ratio	Xiong et al. (2024) Skendi et al. (2020)
Broiler chickens	MOS 0.5%	Improved body weight gain, enhanced intestinal villi morphology, ↑ immune responses	Bełdowska et al. (2024)
Weaned pigs	MOS 0.5–1%	↑ Growth rate, improved nutrient digestibility, ↓ post-weaning diarrhea	Rahman et al. (2022)
Weaned pigs	XOS 0.5%	Modulated gut microbiota, ↑ SCFA production, ↑ feed efficiency	Chen et al. (2021a, 2021b)
Dairy cows	MOS 10–20 g/day	↓ Somatic cell counts in milk, ↑ immune function	Bełdowska et al. (2024) Guerreiro et al. (2018)
Ruminants	XOS 1%	Improved fiber digestibility, potential methane mitigation	Chen et al. (2021b) Guerreiro et al. (2018)
Aquaculture (sea bream)	XOS 0.5–1%	↑ Growth, ↑ resistance to bacterial infection, improved innate immunity	Swanson et al. (2020)

Overall, these data indicate that XOS and MOS are promising feed additives that enhance animal performance and reduce reliance on antibiotics, thereby aligning with sustainable livestock production goals.

## 5 Future perspectives

### 5.1 Personalized nutrition and nutrigenomics

Marked interpersonal variability in responses to dietary fibers and prebiotics suggests that XOS/MOS efficacy is context-dependent, shaped by baseline microbiome, host genetics, habitual diet, and lifestyle. Stratification of responders and non-responders based on microbial composition and functional capacity (e.g., CAZyme repertoires and SCFA production profiles) may improve predictive accuracy. Moving forward, n-of-1 trials and machine-learning models that integrate microbiome features with host metadata should guide dose, DP distribution, and timing of XOS/MOS to optimize glycemic control, lipid modulation, and inflammatory tone. High-resolution readouts (continuous glucose monitoring, digital phenotyping) combined with stool metabolomics may enable closed-loop personalized optimization of XOS/MOS compositions and synbiotic pairings (Zeevi et al., 2015; Shinde and Vamkudoth, 2022; Ali et al., 2025).

### 5.2 Synbiotics

Consensus statements now distinguish complementary versus synergistic synbiotics, with the latter requiring direct evidence that the selected prebiotic preferentially fuels the paired probiotic strain. In this context, rational synbiotic design involving XOS and MOS should prioritize precise substrate-strain matching, taking into account degree of polymerization (DP) windows and linkage specificity that favor target taxa such as *Bifidobacterium adolescentis* or *B. breve* (Feng et al., 2022).

Such designs should be validated through a combination of *in vitro* growth kinetics, cross-feeding network analyses, and *in vivo* colonization and persistence studies. Importantly, future synbiotic formulations will need to demonstrate not only enhanced microbial engraftment but also measurable functional outcomes (e.g., SCFA profiles, metabolic or immunological endpoints) in well-controlled intervention trials to support clinical translation and regulatory acceptance (de Gruening Mattos et al., 2024; Jang et al., 2020; Swanson et al., 2020).

### 5.3 Biorefinery and circular economy

Scaling XOS/MOS sustainably will benefit from biorefinery architectures that valorize agricultural residues (wheat/rice/barley/oat/millet/triticale/buckwheat straw) alongside starch-rich side streams. Priorities include mild pretreatments (microwave/ultrasound/ionic liquids) to preserve oligomer functionality; integrated separations (membranes, SMB chromatography) to deliver narrow-DP fractions; and co-product cascades (lignin for materials/energy; soluble phenolics) to reach favorable techno-economics and near-zero-waste footprints (Norfarhana et al., 2024; Santibáñez et al., 2021; Córdova et al., 2019). Regionally, Kazakhstan’s abundance of residues and enzyme R&D capacity provides a cost-and-carbon advantage for export-grade XOS/MOS.

### 5.4 Enzyme engineering and synthetic biology

Next-generation xylanases, β-xylosidases, α-amylases, and debranching enzymes engineered for enhanced thermostability, inhibitor tolerance, and control of product profile are expected to significantly improve oligosaccharide yields and enable tighter degree of polymerization (DP) distributions. In parallel, synthetic biology chassis can be optimized for high-level enzyme secretion and on-site production, reducing both processing costs and logistical constraints (Kiribayeva et al., 2022, 2024; Mussakhmetov et al., 2024). Furthermore, CRISPR-enabled pathway tuning and directed evolution approaches will facilitate the development of designer XOS and MOS, including arabinosylated XOS and defined MOS fractions with tailored microbiome selectivity. CRISPR-based technologies are increasingly adopted across agricultural and microbial biotechnology, demonstrating high specificity, scalability, and adaptability for strain engineering and functional optimization (Turgimbayeva et al., 2022). These platforms provide a foundation for future pathway tuning in enzyme-producing microorganisms, including those relevant for XOS and MOS biosynthesis.

### 5.5 Multi-omics approaches

Future trials should combine shotgun metagenomics (taxa/CAZymes), metatranscriptomics (active pathways), metabolomics (SCFAs, bile acids, phenolics), and host transcriptomics/epigenomics to map causal chains from structure, microbiota, metabolites, host signaling (Zmora et al., 2019; Cheon et al., 2023). Integrative systems biology will clarify responders vs. non-responders, identify biomarkers of efficacy, and support regulatory dossiers with mechanistic depth in both human and animal applications.

Looking ahead, the field of XOS and MOS research is expected to expand rapidly over the next decade. Future directions include integrating personalized nutrition strategies, developing synbiotic formulations, incorporating them into biorefinery concepts, and applying multi-omics approaches to unravel host–microbe interactions.

**Figure 6:**
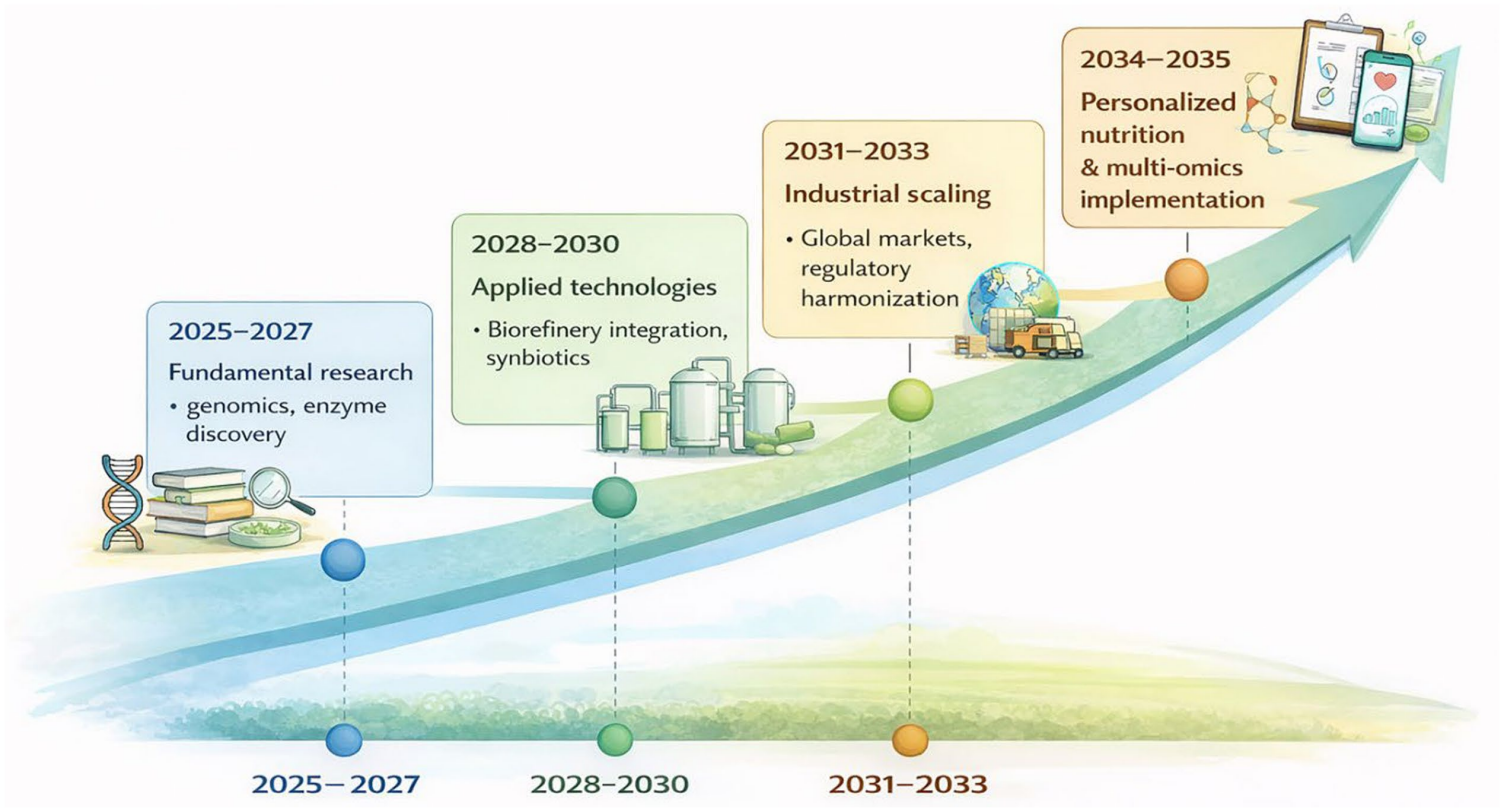
Roadmap of XOS/MOS research and applications (2025–2035).

A roadmap outlining anticipated milestones in XOS and MOS research and applications from 2025 to 2035 is shown in Figure 6.

This roadmap emphasizes a staged progression – from fundamental studies and pilot-scale production to clinical validation, regulatory approvals, and large-scale industrial integration – highlighting the potential of XOS and MOS as next-generation prebiotics in both human and animal nutrition.

## 6 Conclusion

Xylo-oligosaccharides (XOS) and malto-oligosaccharides (MOS) are recognized as leading next-generation prebiotics, attributed to their high physicochemical stability, low effective dosage, and strong selectivity for beneficial gut microorganisms. Substantial evidence indicates that both classes of oligosaccharides provide diverse health benefits, including modulation of glucose and lipid metabolism, regulation of immune function, enhancement of intestinal barrier integrity, and potential protection against chronic metabolic and inflammatory diseases. In animal nutrition, supplementation with XOS and MOS is consistently associated with improved gut health, enhanced growth performance, and greater resilience to enteric stress, underscoring their role as sustainable alternatives to antibiotic growth promoters. Kazakhstan possesses a unique strategic advantage for the development of XOS- and MOS-based technologies, due to its abundant cereal-derived lignocellulosic residues, including wheat, rice, barley, millet, triticale, and buckwheat straw, as well as starch-rich crops such as maize and potato. Coupled with ongoing national advancements in enzyme biotechnology, particularly the development of thermostable xylanases and α-amylases by Kazakhstani research groups, this resource base offers a robust foundation for establishing regional production platforms for functional oligosaccharides.

Integrated and sequential enzymatic conversion strategies provide a flexible technological framework for controlling both the yield and degree of polymerization of XOS and MOS produced from cereal biomass. The strategic combination of pretreatment techniques, customized enzyme mixtures, and product-specific biocatalysts, such as malto-oligosaccharide-forming amylases (MFAs) and xylanases, enables process optimization from maximizing oligosaccharide recovery to targeted synthesis of fractions with specific prebiotic functions.

We propose that the combined application of XOS and MOS may exert synergistic prebiotic effects that surpass those of individual oligosaccharides, driven by complementary fermentation kinetics and broader stimulation of beneficial microbial consortia. Furthermore, we hypothesize that integrating Kazakhstan’s indigenous agricultural biomass with engineered microbial enzyme systems can support sustainable, large-scale production of XOS and MOS with competitive economic and environmental performance.

Progress in this field will rely on the coordinated integration of agricultural feedstocks, advanced enzyme engineering, and scalable downstream processing within unified biorefinery frameworks that adhere to circular economy principles. By addressing human health, animal nutrition, and biomass valorization simultaneously, XOS and MOS are positioned as both promising functional ingredients and strategic cornerstones for advancing global nutrition, sustainable agriculture, and industrial biotechnology.
